# FC-TFS-CGRU: A Temporal–Frequency–Spatial Electroencephalography Emotion Recognition Model Based on Functional Connectivity and a Convolutional Gated Recurrent Unit Hybrid Architecture

**DOI:** 10.3390/s24061979

**Published:** 2024-03-20

**Authors:** Xia Wu, Yumei Zhang, Jingjing Li, Honghong Yang, Xiaojun Wu

**Affiliations:** 1 School of Computer Science, Shaanxi Normal University, Xi’an 710062, China; 2 Key Laboratory of Intelligent Computing and Service Technology for Folk Song, Ministry of Culture and Tourism, Xi’an 710062, China; 3 Key Laboratory of Modern Teaching Technology, Ministry of Education, Shaanxi Normal University, Xi’an 710062, China; 4 College of Computer and Information Technology, Nanyang Normal University, Nanyang 473061, China

**Keywords:** EEG emotion recognition, functional connectivity, GRU, multi-domain feature, attention

## Abstract

The gated recurrent unit (GRU) network can effectively capture temporal information for 1D signals, such as electroencephalography and event-related brain potential, and it has been widely used in the field of EEG emotion recognition. However, multi-domain features, including the spatial, frequency, and temporal features of EEG signals, contribute to emotion recognition, while GRUs show some limitations in capturing frequency–spatial features. Thus, we proposed a hybrid architecture of convolutional neural networks and GRUs (CGRU) to effectively capture the complementary temporal features and spatial–frequency features hidden in signal channels. In addition, to investigate the interactions among different brain regions during emotional information processing, we considered the functional connectivity relationship of the brain by introducing a phase-locking value to calculate the phase difference between the EEG channels to gain spatial information based on functional connectivity. Then, in the classification module, we incorporated attention constraints to address the issue of the uneven recognition contribution of EEG signal features. Finally, we conducted experiments on the DEAP and DREAMER databases. The results demonstrated that our model outperforms the other models with remarkable recognition accuracy of 99.51%, 99.60%, and 99.59% (58.67%, 65.74%, and 67.05%) on DEAP and 98.63%, 98.7%, and 98.71% (75.65%, 75.89%, and 71.71%) on DREAMER in a subject-dependent experiment (subject-independent experiment) for arousal, valence, and dominance.

## 1. Introduction

Emotion recognition is the process of understanding what state of emotion a person is expressing, and plays an important role in various fields, such as neurobiology, medical diagnosis, and artificial intelligence [1,2]. Physiological signals can reflect people’s emotional status truly and objectively, and electroencephalography (EEG)-based methods have shown outstanding performance in accurately identifying emotions [3,4,5,6,7]. However, EEG signals are complicated and complex [8] and contain a certain amount of hidden emotional features. It is a crucial challenge to effectively abstract and integrate various features from EEG to improve the accuracy of emotion recognition.

EEG signals exhibit rich and diverse features across multiple domains, including the temporal, frequency, and spatial domains. These features have been widely used for emotion recognition. Yang et al. considered the time dependence of physiological signals to design a sequence EEG emotion recognition model and achieved an accuracy of 74.4% [9]. Duan et al. proposed differential entropy to represent states related to emotion and achieved a recognition accuracy of 84.22% [10]. Fraiwan et al. used multiscale entropy analysis to extract the mean, slope of the curve, and complexity index of EEG signals to estimate the enjoyment and visual interest levels of individuals, achieving an accuracy of 98% [11]. In addition, studies have reported that different emotions can be successfully captured by EEG spectral differences in various areas of the brain in the alpha band [12], theta band, gamma band, and beta band [2,13]. Moreover, the spatial connectivity relationship between EEG channels has been demonstrated to be associated with emotional responses and has been utilized to enhance recognition accuracy [14]. Obviously, no single-domain analysis can fully reflect the signal characteristics. Furthermore, when the brain processes emotional information, there is often functional connectivity (FC) between brain regions. The FC carries important spatial information that allows people to gain a deeper understanding of how different brain regions coordinate and influence each other [15]. Thus, multi-domain features based on FC should be combined to study emotional status. This approach can efficiently improve the accuracy of emotion recognition.

Recently, many neural network models have been proposed for EEG emotion recognition, among which convolutional neural networks (CNNs) and recurrent neural networks (RNNs) have shown remarkable performance [16,17,18,19,20,21,22]. CNN approaches have significant capability in spatial feature extraction [16,17]. Furthermore, they can directly extract emotion-identifying features from input data by fine-tuning the hyperparameters in the convolutional layer of the CNN [18,19]. Long short-term memory and GRUs, as famous variants of RNNs, are more suitable for processing time series data. They have demonstrated outstanding performance in capturing temporal features for EEG recognition [20,21,23]. GRUs have more advantages than long short-term memory networks, that is, they are simpler, faster, and more efficient [21,22]. Nevertheless, RNNs have limitations in capturing spatial information, while CNNs ignore global information. Since EEG signals contain multi-domain features, single-domain analysis cannot fully represent the complete range of emotional changes. Therefore, inspired by the merits of RNNs and CNNs, we propose a new frequency–spatial high-level feature grasping structure and a multi-domain feature integration strategy based on a hybrid structure of GRUs and CNNs in this paper.

To address these issues, we proposed a temporal–frequency–spatial EEG emotion recognition model based on an FC and CGRU hybrid architecture (FC-TFS-CGRU). FC-TFS-CGRU contains a multi-domain emotional feature extraction module and an attention classification module. In the multi-domain emotional feature extraction module, the phase-locking value (PLV) is utilized to investigate spatial interaction information based on functional connectivity (FC) between brain regions. Subsequently, CNNs are employed to obtain high-level frequency–spatial domain features derived from the combination of PLV and frequency bands. Furthermore, we incorporate GRU networks after the CNNs to capture temporal information associated with the high-level frequency–spatial domain feature, ultimately completing the extraction of multi-domain features based on FC. In the attention classification module, we incorporate an attention mechanism that assigns weights to different features based on their unique contributions to emotion recognition. This integration of captured features improves the accuracy of emotion recognition. The proposed model was evaluated on two popular emotional EEG databases, namely DEAP [24] and DREAMER [25], for both subject-dependent and subject-independent experiments. The results obtained from these experiments demonstrated the proposed model’s superior performance in terms of EEG emotional recognition accuracy across both databases. Our primary contributions are summarized as follows:

1. Incorporating the spatial interaction of brain regions, we introduce the PLV based on FC, which is then used to construct a frequency–spatial matrix with frequency bands to further investigate the elusive high-level frequency–spatial relationship. It significantly enhances the recognition accuracy.
2. A hybrid CGRU architecture is proposed, where a CNN is used to further enhance the frequency–spatial high-level feature extraction, namely, FC-FSH, using the frequency–spatial matrix.
3. GRUs in the hybrid CGRU structure are used to extract the high-level temporal feature of FC-FSH over time. The accuracy of emotion recognition can be effectively improved using the extracted features.

## 2. Materials and Methods

### 2.1. Database and Preprocessing

All EEG signals used in this study were obtained from the DEAP and DREAMER datasets. The DEAP database is a human affective state dataset, where the multi-modal physiological signals of 32 subjects were recoded while they watched 40 videos. After every experiment trial, subjects recorded their emotional state by scoring it from 1 to 9 in four dimensions, namely, arousal, valence, dominance, and liking. The EEG sampling rate was 512 Hz, and the signals were down-sampled to 128 HZ. The number of EEG channels was 32. DEAP can be accessed via the website http://www.eecs.qmul.ac.uk/mmv/datasets/deap/ (accessed on 27 August 2018). DREAMER is a multi-modal human affective state dataset. EEG and ECG signals from 23 subjects were recorded while they watched 18 movie clips. After watching a video, subjects rated the movie using a score from 1 to 5 in three dimensions: arousal, valence, and dominance. The EEG sampling rate was 128 Hz, and the number of channels as 14. The DREAMER dataset can be accessed via the website https://zenodo.org/record/546113 (accessed on 28 July 2023). The details of DEAP and DREAMER are listed in Table 1.

In the preprocessing, signals were down-sampled to 128 Hz, and all the signals in both databases were segmented into 1 s parts [26] with 128 sampling points per window. For DEAP, this resulted in 2400 EEG samples per subject (40 trial × 60 clips), each denoted as $X_{i}=R^{128\times 32}$, where 32 is the number of electrode leads, and 128 is the sample length. For DREAMER, each EEG sample is denoted as $X_{i}=R^{128\times 14}$, where 14 is the number of electrode leads, and 128 is the sample length.

In the label preprocessing, a threshold of 5 was set for the DEAP database and 3 for DREAMER. When the value of arousal (or valence or dominance) was less than the threshold, the corresponding label was set to “low”; otherwise, the label was set to “high”.

### 2.2. The Proposed FC-TFS-CGRU Model

**Figure 1:**
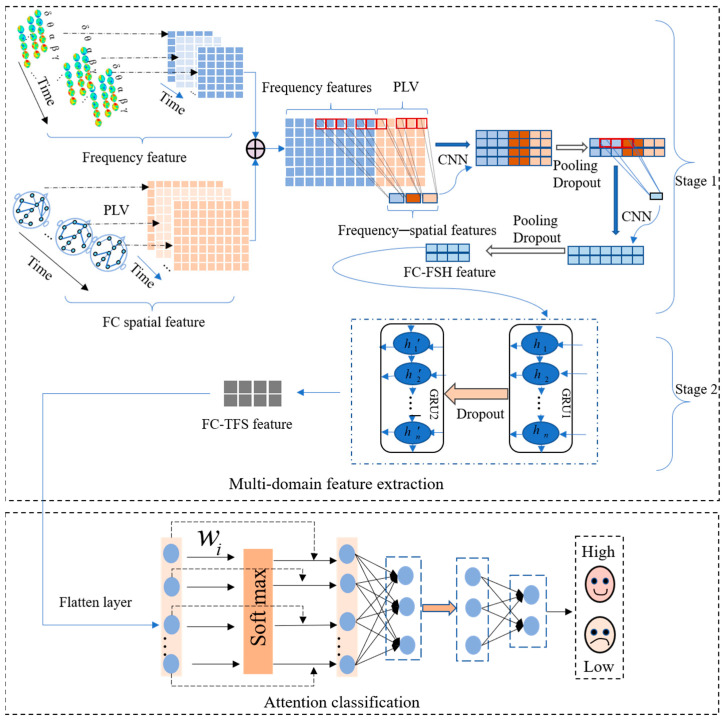
Framework of the proposed model for EEG emotion recognition.

A multi-channel EEG signal is a multi-dimensional time series signal that is rich in temporal, frequency, and spatial features. These features from multiple domains, along with the hidden high-level features among them, all contribute to emotion recognition. Moreover, the extensive interaction information between the channels of multichannel EEG signals is also crucial in revealing the brain’s emotional response, which can be considered to represent emotion recognition. Therefore, to utilize the information to improve the accuracy of emotional recognition, we designed a temporal–frequency–spatial EEG emotion recognition model based on FC and a CGRU hybrid architecture (FC-TFS-CGRU) to recognize emotional states. The FC-TFS-CGRU model is depicted in Figure 1. It significantly enhances the accuracy of emotion recognition by considering the spatial interaction information based on FC and the high-level hidden features across multiple domains.

FC-TFS-CGRU contains two important modules, i.e., a multi-domain feature extraction module and an attention classification module. The multi-domain feature extraction module includes two stages to extract the features in sequence. In stage 1, the phase-locking value (PLV) is used to calculate the spatial features of FC, and Fast Fourier transform is utilized to compose the signal into frequency bands. Then, all of them are combined to further abstract the FC-based frequency–spatial high-level feature (FC-FSH) using the CNN. In stage 2, GRUs are used to abstract the contextual information of the FC-FSH to gain the FC-based temporal–frequency–spatial hybrid feature (FC-TFS), which can reflect the temporal change in the spatial–frequency domain features. Subsequently, an attention mechanism is proposed in the classification process to utilize the different contributions of various features to emotion. The details of each module are illustrated in Section 2.2.1 and Section 2.2.2

#### 2.2.1. Multi-Domain Emotional Feature Extraction

In this section, the multi-domain features of EEG signals will be extracted using the proposed model. The multi-domain emotional feature extraction module comprises two crucial stages: the first is the extraction of FC-FSH, and the second is the extraction of FC-TFS. We will introduce these stages in detail in the following parts.

1. FC Frequency–spatial high-level feature based on CNN

In stage 1 of multi-domain feature extraction, the frequency domain features and spatial features of EEG signals are captured separately, and then, the hidden correlation features between the frequency and spatial domains are further extracted based on these two features. In the extraction of spatial features, unlike most existing studies that consider the physical connectivity between brain regions, we consider the FC of brain regions, which can better respond to the different collaborative relationships of the brain in processing emotional information. The PLV is one of the most important metrics that responds to the FC of the brain, and captures the spatial features based on the FC by calculating the phase synchronization between channels [27]. Thus, there are $N_{C}^{2}$ values of PLV for an EEG signal containing $N_{C}$ channels. Given the symmetry of the EEG signal, $N_{C}(N_{C}+1)/2$ FC spatial features, $F_{s}$, can be obtained. The PLV can be calculated using Equation (1).

(1) $$PLV=\left|n^{-1}\sum_{t-1}^{n}e^{i\left(\phi_{x}\left(t\right)-\phi_{y}\left(t\right)\right)}\right|,$$

where $\phi_{x}\left(t\right)$ and $\phi_{y}\left(t\right)$ are the instantaneous phase of signals x(t) and y(t) in the same trial, respectively. The PLV is in the range [0, 1]. A larger value of PLV indicates a stronger degree of phase synchronization between the two signals.

Frequency domain analysis can accurately reflect the changes in the EEG’s frequency and phase. EEG signals include five different frequency bands, namely, delta 0.5–4 Hz, theta 4–8 Hz, alpha 8–13 Hz, beta 13–30 Hz, and gamma > 30 Hz [28]. Thus, a maximum of $N_{C}\times 5$ band features can be obtained for each EEG signal sample with $N_{C}$ channels. Frequency bands are often found in different brain regions, and spectral changes among varying brain regions are associated with emotional responses. We use these frequency bands to further study the high-level EEG emotion recognition feature associated with the frequency domain. Fast Fourier transform is used to break down EEG signals $x\left(n\right)$ into constituent sinusoids of $H\left(n\right)$ as follows:

(2) $$H\left(n\right)=\sum_{n=0}^{N-1}x\left(n\right)e^{-j\frac{2\pi nk}{N}}k=0,1,\ldots ,N-1,$$

where N is the number of EEG samples and the j is the imaginary unit.

After gaining $F_{s}$ and $F_{f}$, we fuse them to gain a new feature matrix and utilize two CNN layers sequentially to automatically capture their hidden relationship to further study the frequency–spatial high-level features. After every CNN layer, a pooling layer and a dropout layer are connected in series. Therefore, stage 1 contains two convolutional layers, two pooling layers, and two dropout layers. The rectified linear unit (ReLU) function is used as the activation function in the convolution operations. Thus, the input data undergo a convolution operation and an activation operation when passing through a convolution layer. After each convolutional layer, pooling and dropout layers are added to reduce the model size and overcome overfitting; the output after this sequence process can be indicated with the input signal as in Equation (3):

(3) Output=ΦdpPLΦReLuconvInput,(a,b),

where $\Phi_{dp}$ and PL represent the operation in the dropout layer and pooling layer, respectively, ΦReLu is the ReLU function, and (a,b) is the kernel size of the convolutional layer. Finally, after performing Equation (3) twice, the matrix of FC-FSH can be extracted.

2. FC Temporal–frequency–spatial hybrid feature based on GRU network

**Figure 2:**
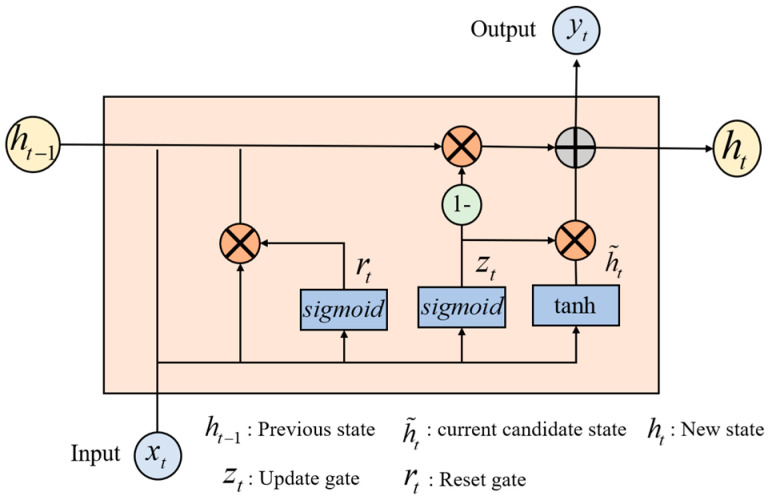
Internal structure diagram of the GRU network.

In stage 2 of the multi-domain feature extraction, the FC-FSH data extracted in stage 1 are used as the inputs to further capture the deep intrinsic correlation features in the temporal–frequency–space domain of the EEG, i.e., FC-TFS. GRU networks have shown effective performance in extracting the long-term dependencies of signals [29]. As shown in Figure 2, the internal structure of the GRU contains two important basic components, the reset gate and the update gate, which control the flow of information. Therefore, GRUs are used at this stage to extract the temporal dependencies of FC-FSH.

When the input signal passes a GRU layer, the new state of the input signal at time t can be calculated as follows:

(4) $$h_{t}=\left(1-z_{t}\right)⊙h_{t-1}+z_{t}⊙{\tilde{h}}_{t},$$

$h_{t-1}$ is the previous state, and ${\tilde{h}}_{t}$ is the current candidate state. The update gate $z_{t}$ decides how much past information to maintain and how much new information to add to the current state $h_{t}$. A larger value of $z_{t}$ indicates that more information about the previous state is brought in to $h_{t}$. $z_{t}$ and ${\tilde{h}}_{t}$ can be obtained as follows:

(5) $$z_{t}=\sigma \left(W_{z}x_{t}+U_{z}h_{t-1}+b_{z}\right),$$

(6) $${\tilde{h}}_{t}=\tanh\left(W_{h}x_{t}+r_{t}⊙\left(U_{h}h_{t-1}\right)\right)+b_{h},$$

where $x_{t}$ is the sample vector at time t and $r_{t}$ denotes a reset gate, which controls how much the previous state contributes to the current candidate state ${\tilde{h}}_{t}$. The smaller the $r_{t}$ value, the smaller the contribution from the previous state. If $r_{t}=0$, then it will forget the previous state. The reset gate is updated as follows:

(7) $$r_{t}=\sigma \left(W_{r}x_{t}+U_{r}h_{t-1}+b_{r}\right),$$

To efficiently determine the temporal relationship of the frequency–spatial domain features, two GRU layers are used in stage 2, and each GRU layer is followed by a dropout layer, which is used to randomly eliminate the connections between the GRU layer and the subsequent connected layers to prevent overfitting. The output after this sequence process can be indicated with the input signal as in Equation (8)

(8) $$Output=\Phi_{dp}\left(GRU\left(\Phi_{dp}\left(GRU\left(Input\right)\right)\right)\right),$$

#### 2.2.2. Attention Classification Module

Electrical signals generated by diverse emotional experiences in humans occur irregularly across various brain regions of the cerebral cortex [30]. Consequently, not all features extracted from EEG signals contribute equally to the classification of emotions. Some features may carry more diagnostic value than others.

Multiple attentional mechanisms have been proposed, drawing inspiration from the brain’s attentional mechanisms. These mechanisms effectively identify the importance of distinct information. Among them, the channel attention system has demonstrated superior performance in exploring information within signal feature maps by directly assigning values to different channels. Hence, inspired by the channel attention mechanism, in this module, we introduce an attention layer following a flatten layer to assign various weights to different features for representing their contributions to emotion recognition. The output formula of the attention layer is shown in Equation (9), where W represents the weight information.

(9) Output=softmax(W·Input)·Input,

After the attention layer, the feature sequence remains in the same dimension and contains weight information. Subsequently, a dense layer and a dropout layer are added to further integrate the features; their dimension is reduced to prevent overfitting. Finally, a Softmax layer is applied to recognize emotional states.

### 2.3. EEG Emotion Recognition Using FC-TFS-CGRU Model

**Figure 3:**
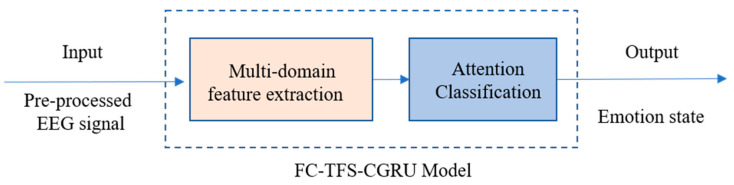
Flowchart of emotion recognition using FC-TFS-CGRU model.

In summary, emotion recognition using the proposed model involves two steps. In the first step, we input the preprocessed EEG signals into the multi-domain feature extraction module, where the frequency bands and PLV of the EEG are calculated initially. Then, the FC-FSH and FC-FSH are extracted in order by the CGRU hybrid structure. After completing the multi-domain feature extraction, the second step includes placing the extracted features into the attention classification module. In this step, the features are converted to a 1D feature sequence with their contribution weights of emotion recognition by a flatten layer and an attention layer. Then, a dense layer, a dropout layer, and a Softmax layer are added in series to classify emotion using the 1D feature. The output of the attention classification module is the final result of the EEG emotion classification using the proposed model. A flowchart of this process is shown in Figure 3.

## 3. Experiment Setup

We utilized the proposed model on subject-dependent emotion recognition and subject-independent emotion recognition on DEAP and DREAMER to verify the performance of our model on EEG-based emotion recognition. All emotion recognition experiments were run in the environment of Python 3.6, TensorFlow = 2.9.0. In addition, some of the figures are drawn by MATLAB2016b.

### 3.1. Implementation Detail

In the subject-dependent experiments, we used 10-fold cross-validation [31] to evaluate the performance of the proposed and baseline methods. Specifically, the average performance of the 10-fold validation process was taken as the final experimental results of one subject, and then, the average accuracy of all the subjects was reported as the final accuracy. In the subject-independent experiments, we adopted the leave-one-subject-out cross-validation strategy to evaluate the EEG emotion recognition performance, where the training and testing data were from different subjects and no information overlap existed. This strategy is also consistent with the experimental settings in [32]. Specifically, in the leave-one-subject-out cross-validation experimental protocol, the EEG data of one subject were used for testing and the EEG data of the remaining subjects were used for training. The experiments were repeated such that the EEG data of each subject were used once as the testing data, and the final classification performance of one emotional label was reported as the average result of all folds. In addition, Adam [33] and cross-entropy were used as the optimizer and loss function, respectively. We set the learning rate of Adam to 0.001, the iteration value to 100, and the batch size to 200.

### 3.2. Performance Evaluation Metrics

The proper quantitative assessment of any deep learning model is crucial in determining its ability to accomplish the intended task. Accuracy and $F_{1}$ are the most common metrics used in classification problems to evaluate the performance of the proposed model [34,35]. For binary classification problems, accuracy and $F_{1}$ can be defined as follows:

(10) $$Accuracy=\left(TP+TN\right)/\left(TP+FP+TN+FN\right),$$

(11) F1=2×Precision×RecallPrecision+Pecall=2TP/2TP+FP+FN,

where TP is true positive, TN is true negative, FP is false positive, and FN is false negative.

## 4. Results

This section details the outcomes of all experiments and analyses the results concisely.

### 4.1. EEG Emotion Recognition Experiments on DEAP

#### 4.1.1. Subject-Dependent Experiment

**Figure 4:**
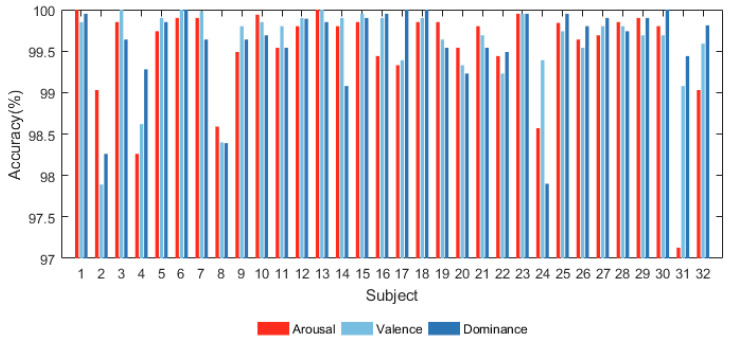
Accuracy of all subjects in DEAP database.

After pre-processing, we utilized the proposed model to categorize every subject’s emotion in the dimensions of arousal, valence, and dominance. The accuracy results on the DEAP database are shown in Figure 4.

Figure 4 shows that the proposed model has significant classification accuracy. Each subject in the DEAP dataset can achieve more than 97% classification accuracy in all three dimensions. Among them, the classification accuracy of several subjects even reaches 1 on several dimensions.

**Figure 5:**
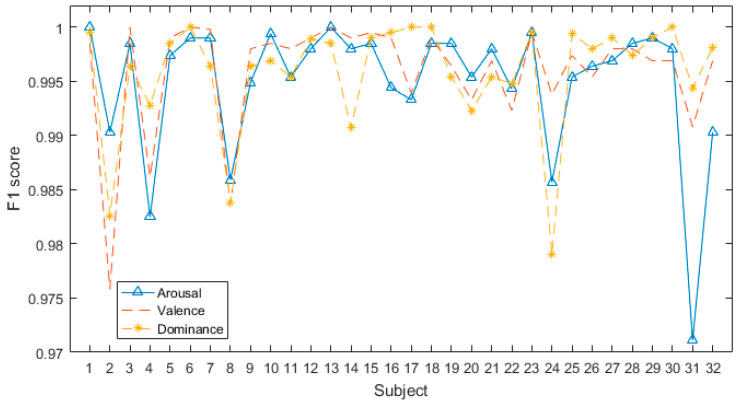
F1-scores of all subjects in DEAP database.

Figure 5 shows that all F1-scores are over 0.97. In the same dimension, the classification accuracy and the F1-score of different subjects are relatively different, which is mainly due to the significant individual differences in EEG signals. Distinct individuals respond differently to the same stimulus material, thereby triggering different emotions.

The mean values of all the subjects’ results were calculated and compared with those of the other models (CapsNet [3], gcForest [4], MLF-CapsNet [36], ATCapsLSTM [5], AP-CapsNet [6], 3DFR-DFCN [37], and ICaps-ResLSTM [38]). The results are shown in Table 2.

Our model achieves the highest accuracies in the arousal, valence, and dominance dimensions at 99.51%, 99.60%, and 99.59%, respectively. It outperforms the least effective model of each dimension by 5.62%, 5.01%, and 4.81%. The comparison of the results illustrates that the proposed model has a significant advantage in EEG-based emotion recognition.

#### 4.1.2. Subject-Independent Experiment

In the subject-independent experiment, each subject was considered as an independent set. Then, the leave-one-subject-out cross-validation was used to divide the training set and testing set, where every subject was used as the testing data and the other subjects were used as the training data. Then, the results of each testing subject were averaged to gain the final classification result. The compared models were FCN-LA [15], JDA-NN [39], BiDANN [40], EEGFuseNet [41], TARDGCN [32], RGNN [42], and GECNN [43]. The results are shown in Table 3.

Table 3 shows that our model outperforms all the compared models with accuracies of 65.74% and 67.05% in the valence and dominance dimensions, respectively. In addition, in the arousal dimension, the accuracy of our model is 58.67%, which is higher than those of FCN-LA, JDA-NN, BiDANN, TARDGCN, RGNN, and GECNN by 3.12%, 4.27%, 4.27%, 0.32%, 7.33%, and 5.7%, respectively. The compared results illustrate that the proposed model can satisfy the requirements of subject-independent emotion recognition and can be effectively applied to emotion recognition for independent subjects.

### 4.2. EEG Emotion Recognition Experiments on DREAMER

#### 4.2.1. Subject-Dependent Experiment

**Figure 6:**
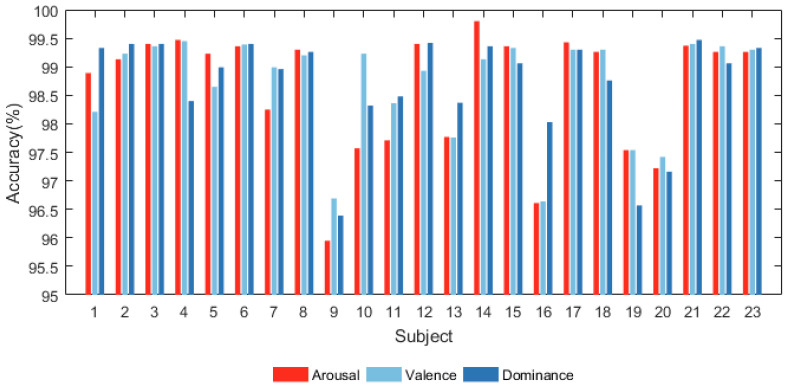
Accuracy of all subjects in DREAMER database.

**Figure 7:**
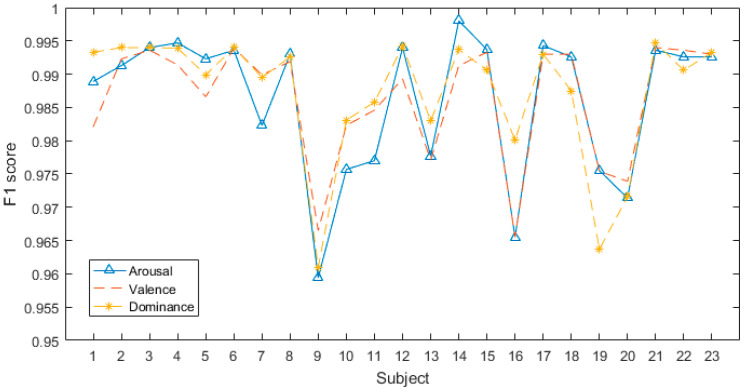
F1-scores of all subjects in DREAMER database.

All the subjects in DREAMER were categorized into the arousal, valence, and dominance dimensions using the proposed model. The results are shown in Figure 6 and Figure 7. Figure 6 shows that the proposed model has significant classification accuracy. Each subject in the DREAMER dataset can achieve more than 95.5% classification accuracy in all three dimensions.

Figure 7 illustrates the corresponding F1-scores of all subjects in the DREAMER database. They are all higher than 0.955, and the highest value can reach 1. The results indicate that our model exhibits high performance in subject-dependent emotion recognition.

To further verify the advantage of the proposed model, we compared it with several emotion recognition models (CpsNet, gcForest, MLF-CapsNet, FP-CapsNet [44], 3DFR-DFCN, GLFANet [45], and ICaps-ResLSTM).

The compared results are shown in Table 4. The findings show that our model outperforms all the compared models, with accuracies of 98.63%, 98.7%, and 98.71% in the arousal, valence, and dominance dimensions. It outperforms the least effective model in each dimension by 8.22%, 9.67%, and 8.82%. The comparison of the results illustrates that the proposed model has a significant advantage in EEG-based emotion recognition on DREAMER database.

#### 4.2.2. Subject-Independent Experiment

For the DREAMER database, the compared models are FCN-LA, JDA-NN, BiDANN, ADDA-TCN [46], HMNN [47], TARDGCN [32], and GECNN. The compared results are shown in Table 5.

Table 5 shows that our model outperforms all the compared models, with accuracies of 75.65%, 75.89%, and 71.71% and 14.54%, 22.19%, and 14.66% higher than the worst model in the arousal, valence, and dominance dimensions. The comparison of the results illustrates that the proposed model can satisfy the requirements of subject-independent emotion recognition and can be effectively applied to emotion recognition for independent subjects.

### 4.3. Network Visualization

**Figure 8:**
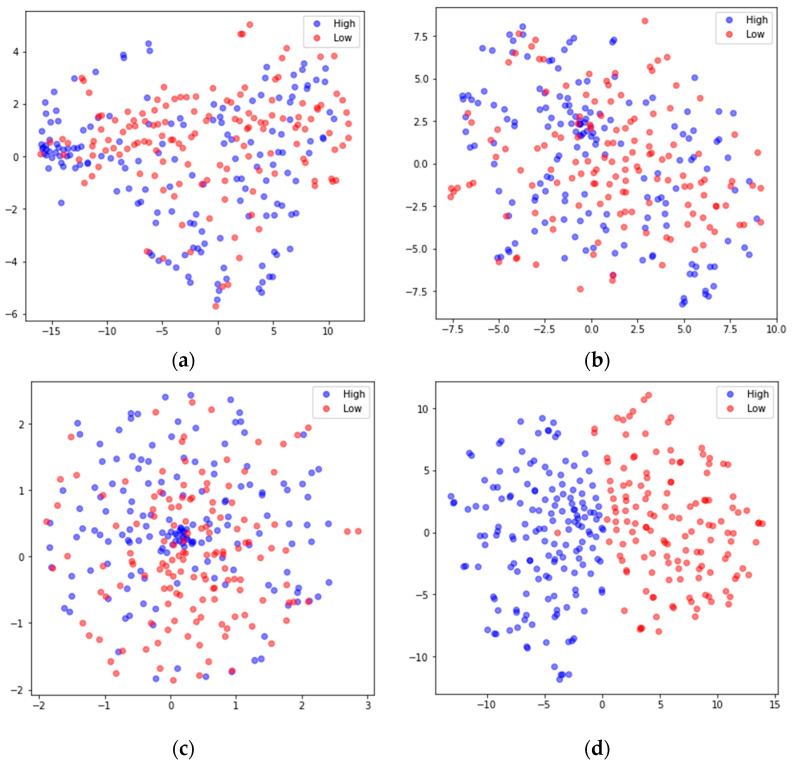
Visualization of feature inputs or outputs of the main layers of the proposed model for subject 1 on DEAP. (**a**) The input of our model; (**b**) the output of the FC-FSH extracted layer; (**c**) the output of the FC-TFS extracted layer; (**d**) the output after the attention and dense layers.

**Figure 9:**
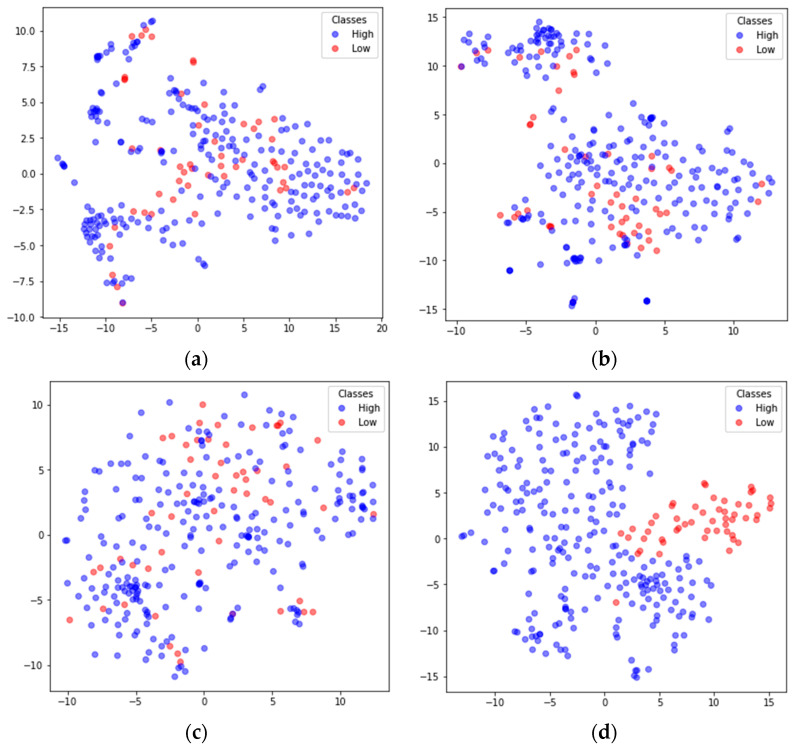
Visualization of feature inputs or outputs of the main layers of the proposed model for subject 15 on DREAMER. (**a**) The input of our model; (**b**) the output of the FC-FSH extracted layer; (**c**) the output of the FC-TFS extracted layer; (**d**) the output after the attention and dense layers.

To better understand the feature extraction capability of the model, the extracted features were visualized using the nonlinear dimensionality reduction algorithm t-SNE [48]. Taking the arousal dimension of S01 from the DEAP dataset and S15 from DREAMER in subject-dependent emotion recognition as examples, the high-dimensional features extracted by the main modules were mapped to 2D features. The results are shown in Figure 8 and Figure 9, where the blue dots represent the feature data corresponding to high arousal (High), and the red dots represent the feature data corresponding to the low arousal class (Low). In both Figure 8 and Figure 9, (a) shows the input feature distributions of the High and Low classes in the arousal dimension, (b) shows the feature (FC-FSH) distributions of the output from the last CNN layer of the FC-TFS-CGRU model, (c) shows the feature (FC-TFS) distributions of the output from the last GRU layer of the FC-TFS-CGRU model, and (d) shows the feature distributions of the output after the attention layer and dense layer of the FC-TFS-CGRU model. In addition, (d) in both Figure 8 and Figure 9 shows that there is almost no misclassification between the two classes, and they can be clearly distinguished.

### 4.4. Ablation Study

The proposed FC-TFS-CGRU method includes three important elements, namely, a PLV feature based on functional connectivity, a CGRU hybrid structure, and an attention classification module. The combination of these three elements leads to the success of the classification tasks. Ablation studies were conducted to further understand which element contributes considerably to the improvement of classification results. For better representation, we used model 1 and model 2 to represent the regular CNN and regular GRU, respectively. Model 3 represents our model without the PLV and attention elements, model 4 indicates our model without the PLV only, and model 5 illustrates our model without the attention element only. The details of all models are presented in Table 6. Then, two ablation experiments were conducted on the DEAP database for subject dependence and subject independence. In all ablation experiments, the signals were segmented by sliding windows with a width of 2 s and moving step of 0.125 s. Five-fold cross-validation and leave-one-subject-out cross-validation were used to evaluate the EEG emotion recognition performance in the subject-dependent experiment and subject-independent experiment, respectively.

#### 4.4.1. Ablation Experiment 1: Subject-Dependent

The ablation results of the subject-dependent emotion recognition are shown in Table 7. All accuracies increased after adding any of the three components of the regular GRU. This finding indicates that all components contribute to the improvement of the EEG-based emotion classification for the subject-dependent experiment. Specifically, as shown in Table 6, the accuracy and F1 of model 3 are higher than those of model 1 and model 2, indicating that the hybrid CGRU structure outperforms the regular CNN and GRU in this task. In addition, the results of comparing model 3 with model 4 indicate that the attention module increases the accuracy and F1 by 0.89%, 0.55%, and 0.47% and 0.89%, 2.29%, and 0.75% for arousal, valence, and dominance. Moreover, the most remarkable increases for the three dimensions are observed when the function connectivity feature is added from model 3, with increments of 9.02%, 8.69%, and 7.48% and 8.89%, 10.64%, and 7.28%. Finally, our model with three parts outperformed model 5, with accuracy increments of 0.51%, 2.57%, and 0.68% and F1 increments of 0.64%, 2.37%, and 1.2% for the arousal, valence, and dominance dimensions.

#### 4.4.2. Ablation Experiment 2: Subject-Independent

Table 8 shows the contribution of different modules to the emotional recognition ability of our model for subject-independent experiments.

Similar to ablation experiment 1, the results in Table 8 show that all components contribute to the improvement of the EEG-based emotion classification results for the subject-independent experiment. Overall, decreases in the accuracies of the three dimensions are observed when the attention module is removed from our model, with decrements of 2.97%, 0, 1.02% for accuracy and 3.49%, 1.28%, and 0.63% for F1. When the PLV is removed from our model, the decrements are 2.4%, 0.4%, and 0.67% for accuracy and 1.59%, 0.68%, and 0.91 for F1. When both the PLV and the attention module are removed, the decrements can reach 3.37%, 2.16%, and 1.6% for accuracy, and 0.25%, 1.34%, and 1.8% for F1.

## 5. Conclusions

In this study, we propose an FC-TFS-CGRU model for EEG-based emotion recognition. A new multi-domain feature grasping method is introduced, and an attention mechanism is integrated to improve the accuracy of emotion recognition. First, we use a PLV to obtain the spatial features of EEG based on the FC of the brain region, and integrate the spatial features with the frequency band features of the EEG to achieve a new feature matrix. Then, a CNN is used to further extract the deep features in the frequency–spatial domain. Second, considering the obtained feature as a sequence, the GRU is introduced. This approach can effectively mine the long-term dependency of the sequence to obtain the temporal information of frequency–space domain features over time, i.e., FC-TFS. Third, during classification, considering the various contributions of different features to emotion recognition, an attention layer is introduced to assign different weights to the captured features, and then, complete the emotional state recognition. Finally, considerable experiments of subject-dependent and subject-independent scenarios are conducted on the DEAP and DREAMER databases to evaluate the performance of the proposed model. The results demonstrate that the proposed feature abstraction method greatly improved the emotion recognition accuracy. Moreover, our model outperforms the state-of-the-art models in EEG-based emotion recognition.

## Figures and Tables

**Table 1:** The detail of the DEAP dataset and the DREAMER dataset.

Type	Description	
	DEAP	DREAMER
Subjects	32	23
Stimulant	Video clips	Video clips
Experiments	40/subject	18/subject
EEG Signals	32	14
Sampling rate	512 Hz	128 Hz
Labels	Arousal, valence, dominance, like	Arousal, valence, dominance
Label scores	1–9	1–5

**Table 2:** Comparison of subject-dependent experiments on DEAP.

Models	Accuracy (%)		
	Arousal	Valence	Dominance
CpsNet	98.05	98.22	98.44
gcForest	97.69	97.53	97.62
MLF-CapsNet	98.31	97.97	98.33
ATCapsLSTM	97.34	97.17	96.5
AP-CapsNet	93.89	95.04	95.08
3DFR-DFCN	95.32	94.59	94.78
ICaps-ResLSTM	98.06	97.94	98.15
Ours	99.51	99.60	99.59

**Table 3:** Comparison of subject-independent experiments on DEAP.

Models	Accuracy (%)		
	Arousal	Valence	Dominance
FCN-LA	55.55	53.63	60.31
JDA-NN	54.4	52.44	59.26
BiDANN	54.4	53.34	56.25
EEGFusNet	58.78	56.27	61.69
TARDGCN	58.35	57.73	61.69
RGNN	51.34	50.12	56.32
GECNN	52.97	51.25	52.95
Ours	58.67	65.74	67.05

**Table 4:** Comparison of subject-dependent experiments on DREAMER.

Models	Accuracy (%)		
	Arousal	Valence	Dominance
CpsNet	94.29	93.94	94.45
gcForest	90.41	89.03	89.89
MLF-CapsNet	95.26	94.59	95.13
FP-CapsNet	95.86	95.48	95.86
3DFR-DFCN	91.3	93.15	92.04
GLFANet	94.82	94.57	94.51
ICaps-ResLSTM	94.97	94.97	94.96
Ours	98.63	98.70	98.71

**Table 5:** Comparison of subject-independent experiments on DREAMER.

Models	Accuracy (%)		
	Arousal	Valence	Dominance
FCN-LA	62.46	60.63	57.05
JDA-NN	65.03	60.55	63.26
BiDANN	63.26	59.98	65.36
ADDA-TCN	63.69	66.56	-
HMNN	64.49	62.51	-
TARDGCN	67.98	61.84	70.28
GECNN	61.11	53.7	57.94
Ours	75.65	75.89	71.71

**Table 6:** Ablation experiment models.

Models	PLV	CGRU	Attention
Model 1	×	×	×
Model 2	×	×	×
Model 3	×	√	×
Model 4	×	√	√
Model 5	√	√	×
Ours	√	√	√

“×” in Table 6 represents the model on the left that doesn’t contain this element, while “√” represents the model that contains this element. For example, Model 1 is a regular CNN model that doesn’t contain PLV, CGRU and Attention.

**Table 7:** Ablation experiment for subject-dependent emotion classification.

Model	Accuracy/F1 (%)		
	Arousal	Valence	Dominance
Model 1	84.31/83.34	82.17/80.16	83.01/83.93
Model 2	86.52/85.02	82.96/84.96	86.72/86.71
Model 3	90.11/90.11	88.42/86.47	91.44/91.44
Model 4	91.0/91.01	88.97/88.76	91.91/92.19
Model 5	99.13/99	97.11/97.11	98.92/98.72
Ours	99.64/99.64	99.68/99.48	99.60/99.92

**Table 8:** Ablation results for subject-independent emotion classification.

Model	Accuracy/F1 (%)		
	Arousal	Valence	Dominance
Model 1	55.12/54.56	62.42/54.24	61.09/62.60
Model 2	55.36/51.94	63.15/52.49	62.94/60.93
Model 3	55.3/56.31	63.58/54.90	65.45/61.46
Model 4	56.27/54.97	65.34/55.56	66.38/62.35
Model 5	55.7/53.07	65.74/54.96	66.03/62.63
Ours	58.67/56.56	65.74/56.24	67.05/63.26

## Data Availability

Publicly available datasets were analyzed in this study. These data can be found here: http://www.eecs.qmul.ac.uk/mmv/datasets/deap/ (accessed on 27 August 2018); https://zenodo.org/record/546113 (accessed on 28 July 2023).

## Abbreviations

CGRU	Convolutional neural network and gated recurrent unit
FC	Functional connectivity
PLV	Phase-locking value
FC-FSH	Functional-connectivity-based frequency–spatial high-level feature
FC-TFS	Functional-connectivity-based temporal–frequency–spatial hybrid feature
FC-TFS-CGRU	Temporal–frequency–spatial EEG emotion recognition model based on an FC-and-CGRU hybrid architecture
